# THE IMPACT OF LEARNING MOTIVATION ON LEARNING WILLINGNESS AMONG OLDER CHINESE ADULTS

**DOI:** 10.1093/geroni/igad104.2123

**Published:** 2023-12-21

## Abstract

As life expectancy increases in China, lifelong learning has become an important approach to successful aging during a long retirement period. In the present study, we examined the association between learning motivation and learning willingness among older Chinese adults. A nationwide sample of 5582 older Chinese adults aged 60 to 93 (Mean age 66.5, SD = 4.67) participated in this questionnaire-based study in 2022. The results indicated that the average willingness to learn of older Chinese adults was 3.14 (range 1-5), with 61.7% of older adults showing an active or neutral willingness for learning in old age. These 3444 older adults with an active or neutral learning willingness were most willing to learn to keep healthy (70.3%) and least willing to learn re-employment skills (19.2%). In terms of learning motivation, these older adults were most likely to regard “make life convenient” as the most important motivation (27.6%). Linear regression showed that after controlling for age, education, social support, physical and cognitive health, stress, self-care ability, and daily activities, motivation could predict learning willingness. Comparing with “make life convenient”, older adults who valued “social comparison” most tended to have less willingness to learn (i.e. choose fewer learning topics, beta = -0.14, 95% Confidence Interval = [-0.23, -0.05]). In addition, older adults who valued “personal growth” (0.30 [0.21, 0.39]) or “alleviate cognitive decline” (0.14 [0.04, 0.24]) most tend to choose various learning topics. Our findings promote an understanding of how motivation is related to the learning willingness of older adults.

